# Trained immunity in farm animals

**DOI:** 10.1186/s13567-025-01594-w

**Published:** 2025-08-08

**Authors:** Marisol Báez-Magaña, Nayeli Alva-Murillo, Alejandra Ochoa-Zarzosa, Joel Edmundo López-Meza

**Affiliations:** 1 Centro Multidisciplinario de Estudios en Biotecnología-FMVZ, Universidad Michoacana de San Nicolás de Hidalgo, Km 9.5 Carretera Morelia-Zinapécuaro, Posta Veterinaria, CP. 58893 Morelia, Michoacán México; 2 Departamento de Biología, DCNE, Universidad de Guanajuato, Noria Alta S/N 36050, Guanajuato, Guanajuato México

**Keywords:** Vaccine, infections, farms, Cattle, goats, pigs, poultry, aquaculture

## Abstract

The mechanisms that enable the innate defence system to “remember its enemies” have opened a new field in immunology, and the evolutionary links among the various defence mechanisms are now being uncovered. In humans, advances in trained immunity have improved our understanding of host–pathogen interactions and broadened the search for new vaccines and treatments as alternatives to antimicrobial drugs. Similarly, veterinary medicine continues to seek strategies to safeguard animal safety, health, and welfare. Animal protein is obtained from meat, milk, or its derivatives, and eggs. Farms play a crucial economic and ecological role, and are a priority for ensuring global food security. The main farm animals used to obtain protein are cattle, sheep, goats, pigs, poultry, fish, and shrimp. Understanding the mechanisms underlying trained immunity in these intensive production systems will deepen our knowledge of host–pathogen interactions and support the development of more effective disease control strategies. It is therefore essential to ensure animal productivity, health, and well-being, as well as to minimise the environmental impact of these intensive production systems through more sustainable practices. This review examines advances in trained and primed immunity in farm animals and discusses the future of trained immunity in the farming sector.

## Introduction

Defence mechanisms are fundamental to the survival of all organisms, from the most primitive prokaryotic cells to complex vertebrates. These mechanisms have evolved to protect organisms from biotic and abiotic stress, ensuring their continued existence and success. Organisms constantly monitor their environment and possess mechanisms that enable them to “remember” recent challenges, allowing for more effective responses to future threats [1].

It has recently been shown that diverse organisms possess an inherent form of immunological memory that operates independently of lymphocytes [2]. Immunological memory is a complex concept with multiple dimensions, often manifested as recognition signals across taxa. However, this may arise from different mechanisms, many of which are independent of the classic immune memory involving B and T cells [3]. Various terms are used to describe this phenomenon (Table 1), including “priming immunity” in invertebrates, “trained immunity” or “innate immune memory” in the innate immune cells of vertebrates, “defence priming” in plants, and “adaptive immune response” for the CRISPR–Cas mechanism in bacteria. Pradeu and Du Pasquier [3] proposed a set of criteria to define immunological memory: strength, duration, speed, specificity, and extinction. They also emphasised that these criteria depend on the phylogeny. In this review, we adopt the definition of trained immunity proposed by Netea et al. to describe the effects of infectious and non-infectious stimuli that enhance the response of innate cells in vertebrate animals [5]. Conversely, we use “immune priming” for invertebrates, as described by Lanz-Mendoza et al. [6] (Table 1).

**Table 1:** **Definitions and concepts used in trained immunity**

Term	Definition/Concept	References
Innate immune memory	Arises from the reprogramming of innate immune cells and other immune-supporting cell types following exposure to a sterile or infectious stimulus. This process depends on the interplay of epigenetic and metabolic changes triggered by the training stimulus. Depending on the nature and intensity of the initial encounter, the modified innate immune response can shift towards a typically anti-inflammatory state (tolerance) or towards a typically pro-inflammatory activation (trained immunity)	[7, 8]
Trained immunity	Exogenous or endogenous insults evoke functional programming of innate immune cells, leading to an altered response to a second challenge after the return to a non-activated state. Trained immunity can be classified into two categories: (1) Long-term memory—occurs when haematopoietic stem cells in the bone marrow are trained, passing pro-inflammatory epigenetic reprogramming to the next generation of cells. (2) Short-term memory—occurs when innate immune cells in peripheral tissues, such as monocytes, macrophages, dendritic cells, natural killer cells, and innate lymphoid cells, are trained	[5, 7]
Tolerance	A host defence mechanism that prevents collateral tissue damage and maintains homeostasis after responding to a pathogen; this strategy is associated with an anti-inflammatory state or a hypo-responsive phenotype	[7]
Adaptive immunological memory	The ability of vertebrates to generate a faster and more effective immune response upon re-encountering a specific antigen, through somatic rearrangement and clonal expansion of lymphocytes	[3]
Priming/immune priming	In invertebrates, immune priming involves an initial exposure of the immune system that triggers a response, which may or may not provide specific protection during homologous or heterologous rechallenge	[6]
Vaccines	Preparation designed to stimulate immune response against diseases	[9]
First challenge	The first exposition of immune cell or organism to a sub-lethal dose of an organism, a vaccine, or metabolic derivatives, followed by a rest period. Examples include endogenous ligands (β-estradiol, oxidised low-density lipoprotein (OxLDL), fumarate, mevalonate, glutamate, vitamin A), and exogenous ligands (BCG, β-glucan, influenza vaccine, measles vaccine, oral polio vaccine, yellow fever vaccine, and COVID- vector ChAdOx1 nCoV-19, and mRNA vaccine BNT162B2)	[10–14]
Second challenge	After the first challenge and rest period, the organism or cell is exposed to a lethal dose of the same or different pathogenic species or strain (i.e. homologous/heterologous challenge), such as *Mycobacterium tuberculosis*, *Escherichia coli*, *Staphylococcus aureus*, *Pseudomonas aeruginosa*, or *Streptococcus pneumoniae*	[4]
Specificity	Indicates whether the secondary response to rechallenge is specific to a particular target (such as a specific pathogen) or exhibits a broad spectrum	[3]

Trained and priming immunity share several similarities, including an enhanced immune response after pathogen stimulation or re-exposure, accompanied by epigenetic and metabolic reprogramming that improves overall immune function. However, there are key differences: (1) In invertebrates, the immune response is based solely on innate defence memory, whereas vertebrates have both innate and adaptive memory responses. (2) In invertebrates, the duration of the response may extend across the life cycle and priming can be transgenerational, passing defence mechanisms to offspring; however, this may come at a cost. For example, priming of the mosquito *Anopheles albimanus* against *Plasmodium berghei* increased survival rates, but was linked to reduced egg hatching rates [15, 16].

While the transfer of antibodies to offspring is well documented in vertebrates, there is also evidence of transgenerational immune priming, particularly in teleost fish, suggesting the possibility of heritable immune training. In addition, training on haematopoietic stem cells in vertebrates provides long-lasting protection by transferring epigenetic marks to the next cell generations [17–20]. Nonetheless, both trained and priming immunity stimulate the innate response, enabling organisms to respond more effectively to pathogen attacks. This is increasingly relevant in the context of drug-resistant pathogens, re-emerging and novel pathogens, and co-infections, all of which are regarded as critically important by the World Health Organization (WHO). The appearance (incidence) of new infections and co-infections is rising as a result of global climate change; for example, the current emergence of the avian influenza H5N1 [7, 10, 21, 22].

In this context, stimulating innate immunity through training or priming could represent a promising strategy against antimicrobial-resistant pathogens, as it offers several advantages, such as enhanced immune responses and protection against pathogens, including re-emerging etiological agents. It is also valuable in the design of new vaccines with improved effects, providing additional protection against pathogens [14, 23]. However, it is crucial to consider potential disadvantages, such as the risk of excessive responses, tolerance, impacts on chronic diseases, limited duration, or reproductive costs in animals. Therefore, a detailed understanding of the cellular mechanisms underlying trained and primed immunity, as well as their downstream effects, is necessary.

Trained and priming immunity have recently been considered as potential strategies to reduce the risk of infections in economically important animals. Many farm animals are at risk of contracting infectious diseases linked to zoonotic pathogens, and preventive measures, such as vaccination, health monitoring, biosecurity protocols, and animal welfare practices, are commonly employed [24]. However, antibiotic overuse and the rise of antimicrobial-resistant pathogens remain major concerns, with global antibiotic consumption in farm animals rising annually. In 2015, the average antibiotic consumption was 45 mg/kg for cattle, 148 mg/kg for chickens, and 172 mg/kg for pigs, exacerbating issues such as antimicrobial resistance [25]. Intensive farming systems, characterised by overcrowding and confinement, further heighten susceptibility to infectious diseases, posing significant health and economic risks. Notable examples include bovine mastitis caused by *Staphylococcus aureus*, influenza A viruses responsible for epidemics in humans and animals, and bacterial infections in fish [26–28].

Emerging infections driven by deforestation, agriculture, and climate change further complicate these challenges. In response, the One Health approach advocates for a balanced strategy that optimises both human and animal health while safeguarding ecological integrity [29]. In this context, trained and priming immunity may offer promising avenues for understanding host–pathogen interactions and for developing effective vaccines to prevent and treat infections in veterinary medicine, thereby contributing to better health outcomes for both animals and humans.

## Trained and priming immunity mechanisms

Trained immunity has been documented in monocytes, macrophages, natural killer (NK) cells, neutrophils, γδ lymphocytes, haematopoietic stem cells, epithelia, and platelets [17, 30–32]. The general mechanism of trained immunity has been investigated primarily in monocytes and macrophages. Following initial interaction with stimuli such as the Bacillus Calmette–Guérin (BCG) vaccine or β-glucan, these cells undergo an epigenetic and metabolic reprogramming. This activation generates a trained immune phenotype, providing enhanced protection against pathogens and increasing the organism’s survival [20, 33, 34].

**Figure 1:**
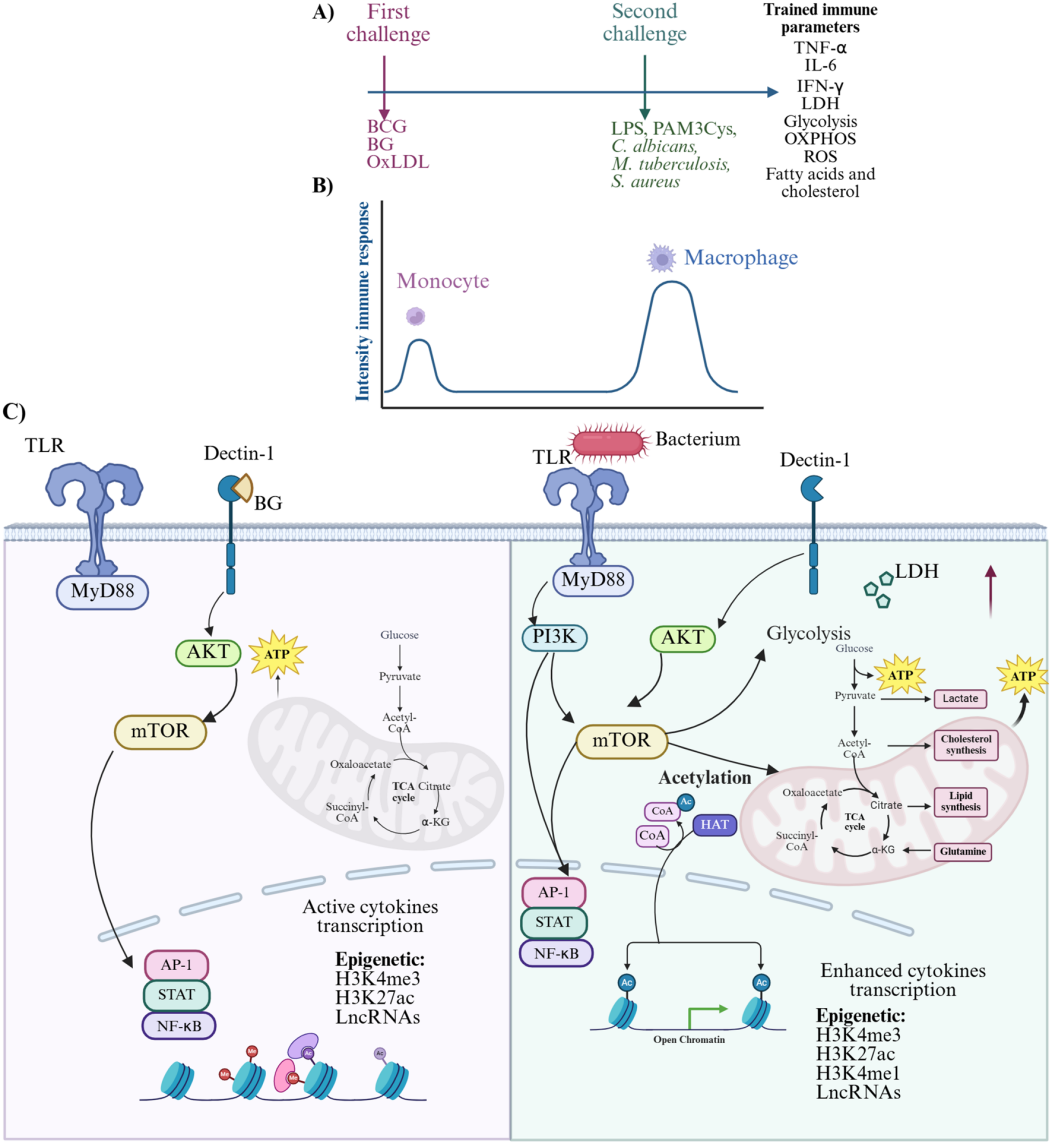
**General mechanism of trained immunity**. **A** Inducers of trained immunity in the in vivo model. **B** Induction of immune response after the first and second challenges. **C** General mechanism induced by β-glucan (BG) as the first challenge. (1) BG is a ligand of the Dectin-1 receptor of monocyte cells; (2) BG-Dec-1 binding activates signalling pathways through Akt and mTOR, leading to metabolic changes such as up-regulation of glycolysis, tricarboxylic acid cycle, OXPHOS, and fatty acid synthesis. (3) This metabolic rewiring subsequently results in epigenetic reprogramming. Key epigenetic marks involved in trained immunity include long non-coding RNAs (lncRNA), histone 3 lysine 27 acetylation (H3K27ac), histone 3 lysine 4 trimethylation (H3K4me3) and histone 3 lysine 4 monomethylation (H3K4me1) in gene promoters. These marks enhance immune activation in response to lethal doses of pathogens or stimuli. Enzymes mediating these marks include Histone-lysine N-methyltransferase 7 (Setd7) and histone demethylase 5 (KDM5) [101, 102]. Image created with BioRender under a publication licence.

Different pathways are activated depending on the antigen; for example, β-glucan binds to dectin-1, which is expressed by monocytes and macrophages, thereby activating the Dectin-1/Raf-1 pathway. In contrast, the BCG vaccine binds to the NOD receptor, triggering the activation of the transcriptional factor NF-κB. Additional signalling pathways are also activated, including Akt, mTOR (mammalian target of rapamycin), and HIF-1α (hypoxia-inducible factor 1α), leading to: (I) a metabolic shift in trained cells characterised by increased glucose uptake and glycolysis pathway induction, tricarboxylic acid (TCA) cycle, OXPHOS, high lactate production, and reprogramming of fatty acid and cholesterol synthesis pathways, and (II) induction of the inflammatory response, marked by elevated production of cytokines (TNF-α, IL-6, IL-1β, and IFN) (Figure 1).

Another fundamental change in trained cells is epigenetic reprogramming [19, 31, 35–37]. These changes include alterations in histones, such as H3K4me3 and H3K27ac, as well as long non-coding RNAs (lncRNAs) and modifications in chromatin architecture. A key point is the link between epigenetic reprogramming and metabolic pathways, which together drive activation in trained cells. For example, fumarate accumulation inhibits the histone demethylase 5 (KDM5), leading to increased trimethylation of H3K4 on the promoter genes of the cytokines TNF-α and IL-6 [3, 11, 20, 33].

In invertebrates, knowledge of epigenetic reprogramming in immune priming remains limited. Immune priming in insects induces epigenetic marks that can persist into subsequent generations, demonstrating immune priming at the intergenerational level (parental to offspring/F1). These epigenetic changes have been linked to alterations in RNA, DNA methylation, and histone acetylation [35]. For example, immune priming induces reprogramming of RNA methylation (5mC) within a single generation, while also inducing DNA methylation and regulating DNMT1 and DNMT2 at the intergenerational level. These changes are associated with sex-specific differences in the expression profiles of immunity-related genes in the offspring. Additionally, histone acetyltransferase (HAT) and histone acetylation mediate maternal intergenerational immune priming, resulting in the specific transcriptional reprogramming of gene expression in F1 larvae [35, 36, 38]. Furthermore, in mosquitoes, immune priming induces metabolic reprogramming that regulates transcripts involved in carbohydrate and lipid metabolism, the TCA cycle, and fatty acid synthesis. Specifically, transcripts encoding trehalose transporter, GDP-D-glucose phosphorylase, and fatty acid hydroxylase were up-regulated [37]. However, the role of epigenetic and metabolic regulation in priming immunity in invertebrates requires further exploration.

## Cattle

There are a few reports related to trained immunity in cattle. Recent evidence indicates that vaccination may induce trained immunity in this species.

The first study to suggest trained immunity in cattle examined monocytes isolated from 1-year-old Holstein heifers (*n* = 6). These monocytes were exposed to BCG (multiplicity of infection; MOI 5:1) as the first challenge. After 6 days, monocytes were restimulated with lipopolysaccharide (LPS, 1 µg/mL) and Pam3CSK4 (10 µg/mL), and cytokine expression was evaluated after 72 h. The results show a significant increase in the mRNA expression of IL-6, approximately two-fold for LPS and four-fold for Pam3CSK4. Similar trends were reported for TNF-α, with approximately two-fold increases for LPS and three-fold for Pam3CSK4 [39]. In vivo effects were also assessed in Holstein bull calves (*n* = 7 animals per group, 6–8 weeks old) inoculated with an aerosol BCG vaccine (10^8^ CFU). Monocytes isolated from vaccinated animals exhibited an increased production of both TNF-α and IL-6 (approximately 20 ng/mL) following challenge with LPS (1 µg/mL). In contrast, Pam3CSK4 (10 µg/mL) significantly increased TNF-α production, but no significant differences in IL-6 were observed 4 weeks post-vaccination. However, trained immunity was not detected in alveolar macrophages [39]. These findings suggest the presence of trained immunity in cattle, which may represent a form of long-term training.

The adaptive immune response in newborn calves is not fully developed, meaning their primary defence mechanism relies on innate immunity. In calves, the immune response involves a high proportion of γδ T cells, which constitute approximately 60% of the total T cells circulating in the bloodstream. These cells, together with NK cells, play essential roles in both innate and adaptive protection, which is crucial for the effects provided by vaccination in calves [40]. The phenomenon of early life protection has been attributed to increased secretion of IFN-γ and IL-12, which help maintain a robust immune response. Furthermore, a correlation between high numbers of NK cells and dendritic cells in neonates has been reported; these data help explain why vaccination in the early stages of life is crucial for a robust immune response [40]. In pre-weaned Holstein calves, two subcutaneous doses of the BCG Danish strain (10^6^ CFU), administered 2 weeks apart, induced functional changes in γδ T cells in response to LPS and Pam3CSK4-1 week after the second BCG dose. These changes included elevated production of TNF-α and IL-6, along with chromatin opening and increased accessibility to genes such as *Siglec14*, *Ifn4*, *Lrrfip1*, and *Tnfrsf10d*; however, mRNA expression was not detected [41]. Additional analyses of epigenetic reprogramming and metabolic modification are needed to confirm immune training.

β-glucans, or β-glucan polysaccharides, are cell wall glucose polymers present in fungi, yeast, algae, and bacteria that are well established to induce trained immunity in humans. The β−1,3-glucan and β−1,6-glucan structures are the most associated with this effect [42, 43]. Recent research indicates that these molecules also induce trained immunity in cattle. Classical (CD14 ^+^ CD16 ^+^) and non-classical monocytes (CD14^-^CD16 ^+ +^) isolated from healthy Holstein–Frisian cows, aged 8.6 ± 3 years, were stimulated with β-glucan (10 µg/mL) from *Saccharomyces cerevisiae*. After 48 h of rest, LPS (10 ng) was added as a second stimulus. Interestingly, the non-classical monocytes showed increased TNF-α production [44]. However, further analyses of cytokines such as IL-1β, IL-6, and IFN-γ are necessary. These findings suggest that the mechanism of trained immunity may be similar in humans and cattle; however, further analysis is needed to investigate epigenetic reprogramming and metabolic regulation [42, 45].

It is also noteworthy that many reports provide insights into potential strategies for inducing cattle immune training (Table 2). For example, male Holstein–Friesian calves, 2 weeks old in field-life conditions, vaccinated with BCG showed a 2.4-fold increase in protection against bovine tuberculosis (*Mycobacterium bovis*) up to 9 months post-vaccination, compared with unvaccinated calves [46]. This study raises the question of whether similar effects could be observed in females, who are economically more important due to their role in milk production. As mentioned, milk is one of the primary sources of protein, making it crucial to understand whether the heterologous effects of vaccines are dependent on the sex of the animal and whether these effects impact milk production. In another study, the heterologous effects of BCG vaccination were assessed on tuberculosis-free 6-month-old Friesian calves. The study investigated whether these effects could be enhanced by using the modified vaccinia virus Ankara strain (MVA85A) or recombinant attenuated adenoviruses (Ad85A). Following the priming effect induced by BCG vaccination, a second priming was generated with the viral vaccine. The combination of both vaccines boosted the immune response of calves, thereby reducing the incidence of *M. bovis* infections [47] (Table 2).

**Table 2:** **Strategies to induce trained immunity in cattle**

Breed	Age	First challenge	Rest	Second challenge	Cytokines	References
Calves (Friesians)	6 months old	In vivo: week 0 BCG (10^6^ CFU/dose) subcutaneous injection; week 8 boost with Ad85A or MVA85A	6 weeks	Intratracheal *M. bovis* (2 × 10^3^ CFU/dose)	IFN-γ, TNF-α	[47]

Holstein–Friesian cows	8.6 ± 3 years old	In vitro*:* monocytes with 10 µg of β-glucan from *S. cerevisiae*	48 h	LPS (10 ng)	TNF-α, IL-6	[44]

Holstein heifers/calves	1–2 years old	**a)** In vitro: BCG (MOI 5:1) in monocytes CD14 ^+^	6 days	LPS (1 µg/mL) or Pam3CK4 (10 µg/mL)	IL-6, IL-1β, TNF-α, RPS9, TLR2, TLR4	[39]
	6–8 weeks old Holstein bull calves	**b)** In vivo: 10^8^ CFU M*. bovis* BCG via aerosol	4–12 weeks	Stimulation in vitro in monocytes: LPS (1 µg/mL) or Pam3CK4 (10 µg/mL)		
	

Another approach involved high-dose BCG vaccination (6 × 10^6^ CFU; contents of two vaccine vials) in 5- to 6-month-old male calves. Vaccination increased mRNA production of pro-inflammatory cytokines TNF-α, CXCL9, CXCL10, IL-17a, IL-12p40, IRF5, and IFN-γ, as well as the enzyme iNOS, potentially contributing to pro-inflammatory immune response after challenge with *M. bovis* [48]. The relevance of this research lies in identifying strategies that can boost the immune responses and improve the health and productivity of economically valuable livestock. A deeper understanding of trained immunity in livestock will support the development of strategies that enhance the quality of life, increase the production of animal-derived products, and contribute to ensuring food security.

## Goat

The first in vivo evidence of trained immunity in goats was reported in newborns administered 50 mg/kg of β-glucan on day 1, with restimulation on day 4. On day 7, LPS was administered (Table 3). Two different β-glucans were tested to induce the first challenge, one from *S. cerevisiae* and the other from *Debaryomyces hansenii*. Only the β-glucan from *D. hansenii* induced a trained phenotype, resulting in increased levels of pro-inflammatory cytokines TNF-α, IL-6, and IL-1β in plasma; nitric oxide (NO) production also increased [49]. In another study, 2-month-old female goats were vaccinated with a heat-killed *Mycobacterium paratuberculosis* Silirum^®^. At 30 days post-vaccination, macrophages were extracted from peripheral blood and differentiated into macrophages over a 7-day period. These cells were then infected with *M. paratuberculosis* at a MOI of 10:1. Macrophages from vaccinated goats showed increased gene transcription of IL-10 and iNOS (1.34- and 2.83-fold, respectively), and were effectively protected against *M. paratuberculosis* [50]. It is also important to note that another small but economically significant ruminant is the sheep. However, evidence supporting trained immunity in sheep is currently lacking. We hypothesise that the possibility of trained immunity may be similar to the mechanism observed in cattle and goats, thus presenting a largely unexplored field of veterinary medicine.

**Table 3:** **Strategies to induce trained immunity in goats**

Breed	Age	First challenge	Rest	Second challenge	Cytokines	References
Saanen–Nubian crossbreed goats	Newborn males	**a)** In vivo*:* β-Glucan, *D. hansenii* or *S. cerevisiae* (50 mg/Kg) orally with 1 mL goat’s milk	2 days	Injection intraperitoneal LPS (12.5 mg/Kg)	IL-1β, IL-6, TNF-α	[49]
	No information	**b)** In vitro: β-Glucan *D. hansenii* (50 mg/ml) monocytes	5 days	LPS (10 ng/mL)		
	
	
Murciano–Granadina goats	2-month-old female	In vivo: subcutaneous injection 2.5 mg/mL vaccine Silirum^®^ (heat-killed 316 F *Mycobacterium avium* subsp. *paratuberculosis* (Map)	48 days	In vitro: *M. avium* subsp. *paratuberculosis* (Map) MOI (10:1)	IL-12, IFN-γ, TNF-α, IL-1β, IL-17A, IL-10	[50]

## Pig

The first report of immune training in pigs was by Byrne et al. [51], who analysed the effects of β-glucan derived from different organisms, including *S. cerevisiae, Candida albicans*, and *Laminaria digitata,* as well as live and inactivated BCG. The results showed that only β-glucan (10 mg/mL) from *C. albicans* increased IL-1β secretion after restimulation with LPS (100 ng/mL) (Table 4). In contrast, other β-glucans even reduced cytokine production, suggesting a response more indicative of tolerance. A trained immunity effect was also observed with BCG (100 mg/mL) for 24 h in porcine monocytes, enhancing production of the cytokines TNF-α and IL-1β after restimulation 5 days later with LPS (100 ng/mL) [51]. These findings demonstrate the potential of BCG to induce heterologous responses in humans and other mammals, such as pigs and cows.

**Table 4:** **Strategies to induce trained immunity in pigs**

Breed	Age	First challenge		Rest	Second challenge		Cytokines	References
White-cross, mixed-breed pigs	< 1 year old	Monocytes: (1) LPS (100 ng/mL); (2) Zymosan (100–0.1 µg/mL); (3) Laminarin (10–0.1 µg/mL); (4) β-glucan *C. albicans* (100–1 µg/mL); (5) IvBCG (100–1 µg/mL); (6) InBCG (100–1 µg/mL)		5 days	LPS (100 ng/mL)		TNF-α, IL-1β	[51]

Landrace x Large White hybrid piglets	10-days-old female	In vivo*:* Oral dose of *M. bovis* (HIMB) 2 mL of 10^7^ heat-inactivated CFU/mL		No information	In vivo: Dose 10^6^ CFU/mL *Salmonella enterica* sub*. enterica* serotype Choleraesuis		TNF-α, IL-1a, IFNγ, IL-8	[52]

Large white/Landrace/Pietrain pigs	3–6 months old	Monocytes or alveolar macrophages: (1) Searup^®^; (2) Ulva extract; (3) Solieria extract	8 days			LPS or Poly:IC	TNF-α, IL-1β, IL-8, IL-6	[53]

Other studies suggest possible strategies or treatments to induce trained immunity in pigs, although more research is needed to support this hypothesis. For example, an immunostimulant based on heat-inactivated *M. bovis* (HIMB) enhances the immune response of pigs, as evidenced by the higher serum concentrations of TNF-α and CCL28 in the immunised group up to 21 days post-immunisation, suggesting that this response may confer protection against *Salmonella enterica* subsp. *enterica* serotype Choleraesuis infections. However, IL-1β and IL-10 serum concentrations decreased from 1 to 21 days post-immunisation. This heterologous protection may be linked to a trained immune response, but further investigation is required [52].

In the search for new treatments to enhance the immune response, plant extracts are being explored as alternatives in both human and veterinary medicine. In this sense, the effects of three algal extracts, Searup^®^ (a commercial *Ulva* extract), non-formulated *Ulva* extract, and *Solieria* extract, were analysed in pigs. Porcine monocytes were treated for 24 h with the extracts, and after a 7-day rest period, the cells were restimulated with LPS or Poly:IC (10 ng/mL each). Following this second stimulus, production of the cytokines TNF-α, IL-1β, IL-8, and IL-6 increased (Table 4) [53]. This provides an intriguing indication of the heterologous effects of alternative approaches to enhance immunity and disease resistance.

## Poultry

To date, studies on poultry have primarily focused on characterising the trained immune response in laying hens and broiler chickens. These investigations provide molecular insights into mechanisms associated with trained immunity and breed differences [54, 55]. The first study was reported by Verwoolde et al. [54], who used monocytes isolated from the peripheral blood of 10-week-old White Leghorn chickens. Training was induced with non-soluble microparticles of β-glucan (M-βG) from *S. cerevisiae* (Table 5). Additionally, they induced the expression of the β-glucan receptor using IL-4. Interestingly, β-glucan triggered immune training in chicken monocytes exposed to LPS (10 µ/mL), an effect that was further enhanced by combining M-βG with IL-4. The results showed that cells exposed to a second LPS challenge increased NO production. Moreover, it enhanced the membrane surface receptors CD40 ^+^ and MHC-II, which are associated with phagocytosis and antigen presentation. This represents the first investigation of trained immunity in birds; however, further research is needed to establish the production of pro-inflammatory cytokines and the potential metabolic and epigenetic mechanisms involved in trained immunity. The full extent of the training’s effectiveness, specifically whether it confers protection against pathogens that commonly affect chickens, also remains to be established [54].

**Table 5:** **Strategies to induce trained immunity in poultry**

Breed	Age	First challenge	Rest	Second challenge	Cytokines	References
White Leghorn chickens	10 weeks old	In vitro: Monocytes β-Glucan *S. cerevisiae* (M-βG, 10 µg/mL); M-βG + IL-4 (100 ng/mL); LPS (10 µg/mL)	6 days	LPS (10 µg/mL)	Not determined	[54]

Ross 308 broiler hens and White Leghorn laying hens	42 days old	In vitro: Monocytes β-Glucan *S. cerevisiae* (M-βG, 10 µg/mL); M-βG + IL-4 (100 ng/mL); LPS (10 µg/mL)	6 days	LPS (10 µg/mL)	IL-1β, TNF-α, IL-10	[55]

Subsequently, Verwoolde et al. compared the trained immune responses in broiler chickens and laying hens. Monocytes were isolated from two breeds of 42-day-old animals. These cells were then stimulated in vitro using a combination of M-βG and IL-4, followed by restimulation with LPS. Layer hen-trained monocytes showed increased NO production and the expression of *IL-1β*, *iNOS*, *arginase 2*, and *IL-10* mRNA, whereas no changes were observed in *TNF-α* expression. Furthermore, expression of the genes *HIF-1* and *PPARγ* increased in trained cells, indicating metabolic shifts in glucose and lipid metabolism. The expression of CD40 and MHC-II further confirmed the induction of trained immunity in layer monocytes. These findings demonstrate that trained immunity can be successfully achieved in laying hens (Table 5) [55].

In contrast, the training was less successful in broiler hen monocytes, as no changes were observed in NO production or CD40 expression. The authors suggest that differences in NO production may be related to age, since it is undetectable in broiler hens at 42 days of age [55]. Further experiments are needed to determine the optimal age and duration for inducing immune training. These results provide valuable insights into the effects of trained immunity across different bird breeds, suggesting that the underlying training mechanisms may involve distinct metabolic changes depending on the breed [55].

## Fish

In fish, the primary model used to study trained immunity is the teleost fish *Danio rerio* (zebrafish) [56–59]. This section discusses evidence suggesting the presence of trained immunity in edible fish.

As noted in previous sections, the BCG vaccine, *Mycobacterium* strains, and glucan are inductors of immune training. The first evidence that *Mycobacterium* strains can induce trained immunity in fish dates back to the 1980s. Modified Freund’s complete adjuvant (MFCA) is a water-in-oil emulsion that contains live attenuated or heat-inactivated strains of *Mycobacterium butyricum* or *M. tuberculosis*. Early studies demonstrated that MFCA administered via intraperitoneal injection in coho salmon (*Oncorhynchus kisutch*), brook trout (*Salvelinus fontinalis*), yellowtail (*Seriola quinqueradiata*), or rainbow trout (*Salmo gairdneri*) conferred protection against *Aeromonas salmonicida*, *A. hydrophila*, *Vibrio ordalii,* or *Pasteurella piscicida.* These findings indicate that the protective effect of MFCA is associated with killed preparations of *M. butyricum* and suggest a trained immune response [60–63].

In fish, the induction of immune training by the BCG vaccine was explored by Kato et al. [64], who proposed that intramuscular administration of the *M. bovis* BCG vaccine confers protection against *Mycobacterium* sp. in juvenile Japanese flounder (*Paralichthys olivaceus*). Gene expression of IL-1β, IL-6, and TNF-α was up-regulated in kidney cells at 1, 3, and 7 days post-BCG vaccination, indicating an immediate immune response. Kato et al. [65] later reported that live attenuated BGC vaccine protects Japanese flounder against *Nocardia seriolae* by a non-specific immune response. This was supported by the up-regulation of gene expression levels of IFN-γ, complement component Bf/C2, and C-type and G-type lysozyme in the kidney of BCG-vaccinated fish. Furthermore, the bacteriolytic activity of serum from BCG-vaccinated fish augmented against *Micrococcus luteus* and *N. seriolae* (1.7- and 2.4-fold, respectively) at 28 days post-vaccination, indicating cross-protection.

In 1990, it was demonstrated that glucan protects Atlantic salmon (*Salmo salar*), rainbow trout, and blue gourami (*Trichogaster trichopterus*) against bacterial infections, including *Vibrio salmonicida*, *Yersinia ruckeri*, *V. anguillarum*, *A. salmonicida*, *Renibacterium salmoninarum,* or *A. hydrophila* [66–69]. The parameters measured in these studies included survival rate, macrophage phagocytic activity, and kidney oxidative burst. The heterologous protection observed may be attributable to trained immunity.

Evidence of trained immunity in catfish has been reported. In this study, channel catfish were challenged intraperitoneally (IP) with β−1−3-glucan (100 µg/g of body weight), and after 1 month, anterior kidney leukocytes were isolated. A second heterologous challenge was then administered (*Edwardsiella piscicida* and *E. ictaluri*) [70]. Higher survival rates (64% and 74%, respectively) were observed in glucan-treated fish at 15 days post-infection (dpi), compared to untreated animals, indicating long-term protection. In vitro, the relative percentage phagocytosis of mCherry: *E. ictaluri* was enhanced in trained catfish neutrophils and macrophages after receiving the second stimulus, which may represent a hallmark of trained immunity. The authors also found that β-glucan increased epigenetic marks, such as H3K3me1, H3K4me3, and H3K27ac, while decreasing H3K27me3 in channel catfish. These modifications were associated with pathways related to phagocytic function, phagocytosis, endocytosis, cell adhesion, and cytoskeletal movements [70].

**Figure 2:**
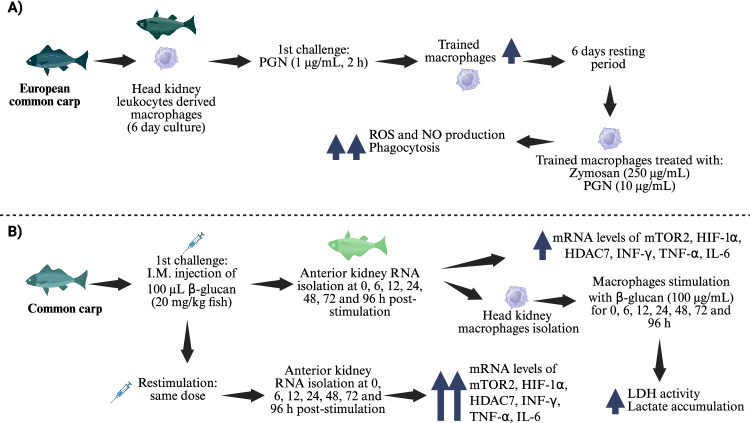
**Representative models of carp-trained immunity**. The innate immune system of carp can be trained with either **(A)** peptidoglycan or **(B)** β-glucan. ADPGK, ADP-dependent glucokinase; ALDH2, aldehyde dehydrogenase. BPGM, bisphosphoglycerate mutase; HDAC7, histone deacetylase 7; HIF-1α, hypoxia-inducible factor 1-alpha; IL, interleukin; LDH, lactate dehydrogenase; mTORC2, mammalian target of rapamycin complex 2; NO, nitric oxide; PGN, peptidoglycan; ROS, reactive oxygen species; TNF-α, tumour necrosis factor-alpha. Vertical blue arrows indicate levels of up-regulation. [71, 72]. Image created with BioRender under a publication licence.

In contrast, carp macrophages were trained in vitro with NOD-ligand peptidoglycan (PGN) for 2 h, then rested for 6 days. At that point, the cells were treated with heterologous (zymosan) or homologous (PGN) stimuli (Figure 2A). Interestingly, reactive oxygen species (ROS) production increased in zymosan- or PGN-stimulated trained macrophages compared with unstimulated untrained macrophages. However, NO production was elevated only in zymosan-stimulated trained cells. In addition, there was up-regulated expression of some genes of cytokines (*IL-6*, *TNF-α*, *IL-1β*), and metabolic enzymes (*adpgk*, *aldh*, *bpgm*) in unstimulated trained macrophages, along with increased lactate production [71]. In another study, Waikhom et al. [72] provided evidence of trained immunity in β-glucan-challenged carp macrophages (Figure 2B). Mature common carp were stimulated intramuscularly with β-glucan (20 mg/kg fish) and rested for 6 days. The carp were then restimulated with the same dose of β-glucan, and fish were collected at 0–96 h. Transcript levels of trained immunity markers (*INF-*γ, *IL-6*, *TNF-α*, *mTORC2*, *HIF-1a*, and *Hdac7*) were up-regulated in the anterior kidney tissue of β-glucan-stimulated carp; these levels increased after restimulation, as did lactate dehydrogenase (LDH) activity and lactate concentration, compared with β-glucan-stimulated cells.

Waikhom et al. [73] evaluated markers of trained immunity in β-glucan-trained Nile tilapia (*Oreochromis niloticus*) using both in vitro and in vivo approaches. Fishes were injected IP with β-glucan (200 µg/10 g fish; stimulation group). Another group received the same dose of β-glucan, and after a 6-day resting period, was restimulated with β-glucan (restimulation group). A control group was treated with PBS [73]. Fourteen days post-treatment, Nile tilapia were infected IP with *Streptococcus agalactiae*. The survival rate was higher (60%) in the restimulation group than in the stimulation or control groups (46.66% and 26.66%, respectively). Markers of trained immunity (*IL-6*, *IL-12*, *TNF-α*, *INF-γ*, *mTOR*, *HIF-1α*, and *Hdac11*) were analysed in head kidney tissue from primed tilapia before infection. Overall, the relative gene expression of cytokines was consistently elevated from 0 to 72 h in both stimulation and restimulation groups, with higher levels observed in the latter. A similar trend was observed for the relative expression of *mTOR*, *HIF-1α*, and *Hdac11*. In addition, LDH activity and lactate production were measured in β-glucan-trained macrophages, and both parameters were up-regulated from 0 to 96 h. These results suggest that trained immunity may enhance the health of Nile tilapia.

It has been reported that β-glucan and zymosan protect turbot (*Scophthalmus maximus*) against infection by *A. salmonicida* or *E. piscicida* [74, 75]. The survival rate of juvenile turbot trained either with β-glucan (0.05–1.25 mg, IP injection or immersion) or zymosan (0.05–0.25 mg, IP administration) and then infected IP with *A. salmonicida* was between 30 and 40% (13 dpi), compared to 100% mortality in fishes infected with bacteria. However, when fish were trained with β-glucan plus 2-deoxy-D-glucose (2-DG, a glycolysis inhibitor) and then infected, no protection was detected, suggesting that metabolic alterations, often associated with trained immunity, underlie the effects of β-glucan. A longer priming effect was also observed, as 40% of turbot survived after 25 days of β-glucan administration (11 dpi). When turbot were trained with β-glucan (0.1 mg, IP, followed by a 7-day resting period), and then challenged with *E. piscicida* by immersion, a higher survival rate was recorded at 30 dpi compared to infected fishes. Additionally, lower bacterial loads were recovered from the posterior intestine, liver, spleen, and head kidney at 7 dpi [75]. Inflammatory cells were detected in the head kidney and spleen of infected trained fish, alongside up-regulation of *IL-1β*, *IL-6*, and *TNF-β* relative gene expression in the gills, liver, and spleen of β-glucan-trained fish from 1 to 3 dpi, as well as increased *mTOR* and *HIF-1α* mRNA levels in trained, infected turbot. Using scRNA-seq technology, the authors suggested that neutrophils may be key effector cells of β-glucan-trained immunity in turbot. This training elevated the expression of genes codifying pro-inflammatory cytokines (*IL1R1b*, *NFKbiaa*, *RIPK2*, and *TGFb1a*), epigenetic enzymes (*ep300b*), and transcription factors (*rbpj*, and *stat3*) in infected-neutrophils, as well as the IL-1R signalling pathway, ROS, NO, and lactate production, myeloperoxidase (MPO) activity, and neutrophil extracellular traps (NETs), compared with the control group. These findings indicate that β-glucan can induce heterologous protection against bacterial infection, with neutrophil IL-1R signalling-regulated metabolic reprogramming playing a crucial role in NET formation and bacterial killing in turbot.

Other research has demonstrated the protective effect of glucan against viral infection [76]. In this study, head kidney macrophages were isolated from healthy sevenband grouper and treated with β-glucan (5 µg/mL, 24 h). After 6 days of resting, the cells were infected with nervous necrosis virus (NNV, 1 h) and monitored for up to 72 h. The results indicated virucidal activity in macrophages during the later stages of infection, as evidenced by a decrease in viral copies in the trained group compared to naïve cells. ROS and NO production were enhanced in trained macrophages, although infection reversed this effect. In addition, mRNA levels of pro-inflammatory cytokines (IL-1β, TNF-α, and IL-6) were down-regulated in infected trained macrophages compared with NNV-infected control cells. The opposite effect was observed in mRNA levels of HIF-1α (a marker of trained immunity) and IFN-1 (antiviral immunity). Elevated lactate concentrations at 3 dpi were detected in NNV-infected trained macrophages, suggesting enhanced glycolysis following training. Furthermore, in vivo, fish were immunised IP with β-glucan (10 µg in 100 µL of PBS), and after 14 days post-stimulation, sevenband grouper showed protection against a secondary challenge with NNV.

Further investigation is needed to understand the contribution of trained immunity in protecting fish, including its potential for cross-protection. It is crucial to determine the optimal administration route (oral, IM, IP, immersion), the appropriate physiological state (juvenile or larval), and whether adjuvants are necessary to induce an adequately trained immunity, as well as to elucidate the underlying molecular mechanism. Moreover, additional research is needed to investigate the duration of protection following the induction of trained immunity.

## Shrimp

Although invertebrates lack an adaptive defence system, their innate immune system is sufficient to recognise and eliminate pathogens. Since the late twentieth century, evidence has indicated that the shrimp innate immune system is capable of priming to elicit improved and long-lasting protective responses against pathogens as “immunological memory”. This memory is attributed to the plasticity of the immune system [6]. The shrimp immune system shares features with the vertebrate innate immune system, including pattern recognition receptors (such as Toll-like receptors, TLRs) and both humoral and cellular components that contribute to priming immunity or trained immunity, as seen in vertebrates [77–79]. In these studies, improvement of the shrimp immune system through ‘vaccination’ against primary pathogens employed various administration routes, including IM injection, immersion, spraying, and oral delivery (via enriched brine shrimp *Artemia salina*) [78, 80, 81]. However, injections appear to be the least suitable method for mass immunisation of pond shrimp.

At least five haemocyte subpopulations have been identified in *Penaeus vannamei*, which are analogous to vertebrate immune cells, each one with specific functions in pathogen recognition and phagocytosis [82]. This diversity has been linked to the ability of formalin-inactivated *Vibrio harveyi* vaccination to induce priming immunity in young shrimp [83]. Priming immunity has also been induced using formalin-inactivated *Vibrio* sp., a commercial vaccine preparation (AquaVac Vibromax), heat-killed *Vibrio alginolyticus*, biofilm from *V. alginolyticus*, LPS from *V. harveyi*, recombinant VP28 protein from white spot syndrome virus (WSSV, lvp28), inactivated virus, recombinant DnaK, and *Pandanus tectorius* leaf extract [78, 83–88]. These studies indicate that the protective effect of immune priming is evident when challenges with the corresponding pathogen are conducted. Additionally, some studies have incorporated immunostimulants to enhance vaccine efficacy, such as probiotics (gamma-irradiated *V. parahaemolyticus*) [89] and carboxymethyl β−1,3-glucans (CMBG) [90, 91].

The immunological parameters measured in the studies included the phagocytosis index; activities of enzymes such as prophenoloxidase (proPO), superoxide dismutase (SOD), lysozyme, and alkaline phosphatase (ALP); gene expression of immune elements (TGase-a, lvHSP70, crustin, proPO); quantification of superoxide anion; haemolymph antibacterial assay, and haemocyte counts [78–81, 84, 85, 87–91]. Survival or mortality rates, along with viral or bacterial loads, were also recorded. However, not all these parameters were assessed in every study.

Understanding the mechanisms of immunological memory in invertebrates is crucial for developing strategies to improve shrimp immune defences against pathogens and to advance the development of vaccines and immunological therapies. Furthermore, this knowledge may provide key insights into the evolution of immunological memory, from invertebrates to vertebrates.

## Future of training immunity in farm animals

A major challenge facing veterinary medicine and animal production is meeting the global demand for food while ensuring safety standards and minimising environmental impact. This is a complex issue that requires addressing multiple challenges simultaneously. However, this review highlights research in veterinary immunology, specifically focusing on enhancing animal immune responses through prophylactic measures. Such approaches can significantly reduce the need for therapeutic interventions, thereby contributing to the overall goal of sustainable and ethical farming practices.

The history of trained immunity in animals is only just beginning to be written; there are still relatively few reports in this area, and further research is needed to establish appropriate treatments. Based on current findings, even humans and mice have considerable ground yet to cover. Veterinary medicine encompasses a wide range of species, making it challenging to generalise conditions and treatments. As described, a molecule such as β-glucan from *C. albicans* is capable of inducing immune training in humans [45], while in calves, similar effects are achieved with the β-glucan from *S. cerevisiae* [92]. In goats, however, β-glucan must be sourced from *D. hansenii* as *S. cerevisiae* does not induce training [49]. These observations suggest that the origin of the molecules is crucial in generating the trained immunity phenotype and may also be linked to the environmental conditions in which the individual develops.

The future of trained immunity may focus on understanding the relationship between the environment and immune response. This relationship has already been studied in humans [93]. Seasonal changes and environmental temperature significantly influence immune response and susceptibility to specific pathogens. In livestock, it has likewise been observed that animals are more vulnerable to diseases at certain times of the year. Therefore, elucidating the role of the innate immune response, environmental effects, and seasonal vulnerability is crucial for developing new strategies to protect against disease. It is also important to investigate the impact of trained immunity in milk and meat production, and its relationship with seasonal variation, genetic factors, and diet [94]. Addressing these questions will require detailed epigenetic analyses.

The demand for food and protein sources has risen markedly, and globalisation has facilitated access to a broader range of foods. As a consequence, fish farming and seafood production have expanded significantly. However, this intensive exploitation of aquaculture has also led to an increase in disease prevalence and a greater reliance on antimicrobials. A responsible approach must incorporate ecological and sustainable alternatives. One strategy is to deepen our understanding of immune mechanisms and to develop new therapies. This review highlights vaccines and molecules capable of inducing a trained immunity phenotype. To develop preventive and sustainable measures for controlling disease, it is essential to understand the molecular mechanisms that occur during training in fish and shrimp.

In the context of fish aquaculture, it has been suggested that certain molecules, including Freund’s complete adjuvant or MCFA, the peptide FK-565 (heptanoyl-γ-D-glutamyl-(L)-mesodiaminopimelyl-(D)-alanine), glucan diet, vaccines against VHSV (viral haemorrhagic septicaemia virus) and IHNV (infectious haematopoietic necrosis virus), prolactin, and LPS from *A. salmonicida*, have the potential to induce trained immunity in fish [60–72, 95–100]. This is supported by evidence showing that these molecules confer cross-protection against bacterial or viral infections, enhance survival rates, and induce oxidative bursts. These findings underscore the potential for identifying molecules capable of inducing trained immunity.

The intake of a diet rich in glucan by fish could represent a future strategy for protecting aquaculture species against bacterial infections and for enhancing their innate immune response. This is supported by reports in Nile tilapia and juvenile orange-spotted grouper (*Epinephelus coioides*). For example, Nile tilapia fed MacroGard^®^ (0.1%; β−1,3- and β−1,6-glucans from *S. cerevisiae*) showed increased production of O_2_^−^, as well as elevated complement, lysozyme, and MPO activities; glucan also protected fish against infections with *Aeromonas sobria* and *S. agalactiae* [99]. In orange-spotted grouper, diets supplemented with a mushroom β-glucan mixture (MBG; 0.5 g, 1 g, 2 g, and 1 kg) led to an increase in phagocytic, lysozyme and alternative complement activities, and enhanced respiratory burst after 6–30 days, compared with untreated fish [100]. Furthermore, when these groupers were challenged with *V. alginolyticus*, survival rates were higher in juveniles trained in 1 or 2 g MBG. This review also discussed the effects of plant-derived β-glucans, such as those from *Ulva* spp. (green seaweed) and laminaran from brown algae as inducers of trained immunity [53, 67]. These findings are promising for developing innovative formulations of supplemented feed for farm animals, providing a more accessible approach to enhancing immune responses. They also establish a foundation for further exploration of new β-glucan sources that may serve as immunological modulators.

Future perspectives of trained innate immunity also include exploring vaccines already used in farm animals. Another important consideration is tolerance. It would be valuable to examine the response according to the sex, breed, life stage, and the influence of the environmental factors, among others, as this could guide the design of more appropriate vaccination strategies, not solely based on antibody production, while seeking to avoid tolerance effects. An interesting approach to enhance vaccine effectiveness is the search for adjuvants that can improve heterologous responses. There is also a growing interest in discovering immunomodulatory molecules and investigating plant-based sources as complementary treatments to booster the immune response. These combined efforts hold significant promise for advancing the field of immunology. All these considerations also involve the animal’s own physiology and the cellular mechanisms. This information provides a foundation for understanding the mechanisms at work in animals. The depth of such findings will help clarify the scope of infections and treatments, as well as potential unwanted effects related to immune training. Although genetic improvement of animals was not addressed here, selecting the most immunologically robust animals to strengthen the breed may represent a new application of trained immunity. Ultimately, these new alternatives to antimicrobials, together with enhancements to the animal immune response, can reduce factors that affect the environment and positively support improved access to animal-derived protein, thereby ensuring food safety.

## Conclusion

Intensive farming practices in both terrestrial and aquatic environments expose animals to bacterial, viral, and parasitic infections. Antimicrobial agents are often used to maintain production levels, which can lead to the emergence of resistant strains and the development of new pathogens. Consequently, the escalating use of antimicrobials compromises food safety, animal health, and human health. Moreover, the rise of antimicrobial resistance in animals and humans can complicate the treatment of infectious diseases, particularly in immunocompromised individuals. In this context, trained immunity offers a relevant and sustainable prophylactic alternative in veterinary practice, helping to control infectious diseases while supporting food security and global access to animal protein.
